# Algae as a potential source of protein meat alternatives

**DOI:** 10.3389/fnut.2023.1254300

**Published:** 2023-09-07

**Authors:** Johanan Espinosa-Ramírez, Alicia C. Mondragón-Portocarrero, Jose A. Rodríguez, Jose M. Lorenzo, Eva M. Santos

**Affiliations:** ^1^Tecnologico de Monterrey, School of Engineering and Sciences, Monterrey, Mexico; ^2^Laboratorio de Higiene, Inspección y Control de Alimentos, Departamento de Quimica Analitica Nutricion y Bromatología, Universidad de Santiago de Compostela, Lugo, Spain; ^3^Área Académica de Química, Universidad Autónoma del Estado de Hidalgo, Pachuca, Mexico; ^4^Centro Tecnológico de la Carne de Galicia, Ourense, Spain

**Keywords:** microalgae, macroalgae, seaweed, meat analogs, bioactive peptides

## Abstract

With the rise of plant-based meat alternatives, there is a growing need for sustainable and nutritious sources of protein. Alga is a rich protein source, and initial studies show that it can be a good component in developing protein meat alternatives. However, there are certain limitations in their use as the need for efficient and optimal technical process in large-scale protein extraction and purification, as well as overcoming certain negative effects such as potentially harmful compounds, allergenicity issues, or sensorial affections, especially in color but also in textural and flavor characteristics. This review offers a vision of the fledgling research about using alga protein in the development of meat alternatives or supplementing meat products.

## 1. Introduction

Food production is one of the major worldwide concerns because food demand will increase in the next decades. In this regard, animal and plant protein sources must be aligned with consumer requirements. Animal-based proteins, dairy and meat mainly, have been the most consumed protein source. However, they are facing several inquiries related to sustainability issues as well as negative effects on human health. Plant-based proteins have attracted the attention of a sector of the population interested in low-fat and vegan diets, with high concern about renewable and sustainable alternatives for animal-based proteins (1). In this sense, this growing demand should consider not only the sustainable production process of the protein but also without compromising nutritional and safety factors. Soy, legumes, and oil seeds have been the first targets in the search for plant protein sources (2). However, novel sources like algae proteins, a non-animal protein have been considered as a relevant alternative. Algae have a high growth rate, high photosynthetic efficiency, and low water consumption, and they do not require land for growth (3).

Algae can be defined as a group of autotrophic photosynthetic, aquatic, and non-embryophytes organisms. They can be unicellular, colonial, present filaments, or be composed of simple tissues (4). It is usually to find the terms microalgae and macroalgae (commonly called as “seaweeds”). Microalgae are microscopic prokaryotic or eukaryotic photosynthetic microorganisms, they are typically unicellular or simple multicellular organisms, only visible with magnification, which grow and live in suspension in water columns (5). For human consumption, the microalgae *Arthrospira, Chlorella, Dunaliella*, and *Haematococcus* have been approved by the European Food and Safety Authority (6). Microalgae proteins are dried cells that are usually sold as protein supplements, although they can contain some bioactive compounds (7).

On the other hand, macroalgae are photosynthetic organisms visible to the naked eye and with more complex multicellular forms. Some have plant-like structures, usually growing on the rocky bottom of coastal waters. They can be classified according to their pigmentation: brown (*Ochrophyta*), green (*Chlorophyta*), and red (*Rhodophyta*) (4). In Asia, macroalgae have been included in the human diet for centuries and have been used for hydrocolloid extraction (alginate, agar-agar, and carrageenan), habitual ingredients in food processing industries.

Microalgae can achieve a protein content higher than 70% while macroalgae contents use to being lower (9–22%) in dry matter. In both cases, the main advantage is their essential amino acid contents. The high protein content and good amino acid composition are the main algal protein highlights compared to other plant-based proteins, such as soy or chickpea (8, 9). Despite their advantages, the development of algae-based meat alternatives is still in the initial stages. This review presents recent advances in algae research for their possible inclusion in meat products including the technological and safety challenges.

## 2. Protein extraction from algae

### 2.1. Protein contents in micro and macroalgae

Nowadays, the need for a protein transition from animal foods to other sources is evident. The world population is increasing continously, and the production of food animals as the main protein source presents serious drawbacks derived from its high carbon footprint (10). Due to the health issues related to meat intake, there is also a growing trend toward reducing meat consumption through a shift to consuming more plant-based or plant-forward diets and foods (11). In fact, veganism is gradually becoming a widespread phenomenon not only in Western societies but across cultures around the world (12). Among the potential alternatives to animal protein sources, algae have important advantages over terrestrial vegetables, as they do not require arable land, freshwater, or artificial fertilizers, and have high productivity (13).

One potential source of non-conventional proteins is seaweed. Protein content represents between 19 and 47% in dry weight in red algae, while green and brown algae present lower protein amounts, around 10–20% in dry weight (14, 15). These values are comparable to, or are even higher, than those contained in other terrestrial plant sources such as legumes (20–30%), cereals (10–15%), or nuts (20–30%) (15), and even higher than those of some traditional animal protein sources such as milk (13). With respect to microalgae, in the biomass resulting from microalgae cultivation, proteins represent between 3 and 36%, with most species having values close to 40% (16), depending of the species and growth conditions, although some species, such as *Arthospira platensis* can contain even up to 70% of protein (17).

For all these reasons, and given that seaweed cultivation does not compete with terrestrial vegetable crops and the natural resources that are necessary for this crop, the increase in the production of proteins from algae could contribute to a more sustainable dietary consumption (14). Although nowadays, most of the seaweed production in the world still comes from the harvesting of wild seaweed, its cultivation has increased markedly in the last decades (10). In recent years, there has been an increasing trend in the production of algae, with red seaweed (*Rodophyta*) being the most cultivated, accounting for more than 50% of the total (15), which is the genera with a higher protein content (14).

However, the food industry's utilization of proteins from seaweed protein as an ingredient presents some relevant drawbacks. One of the main concerns of using seaweed as a protein source is the large variability in the protein content in the raw material, which can vary from 3 to 47% (18). Such variation relies on several parameters, such as algal species, harvesting season, and location. For example, some algae have been reported to contain more protein in winter and early spring, while protein is lower in summer and autumn (19). In addition, previous studies have revealed that cultured seaweeds have shown higher protein content, regardless of the protein analysis method (20). This situation presents a challenge given the crucial role of standardizing raw materials in the food industry to maintain homogeneity across different produced batches (22). Algal proteins are usually cross-linked to polysaccharides via disulfide bonds within the cell wall assembly limiting their extraction (10). Due to this complex structure, the employed protein extraction methods rarely achieve protein yields over 50% of the total protein (15), and present a high variability, which complicates the standardization processes (22).

Due to the great variability in protein contents and extraction yields, adequate methods for quantifying proteins are essential (15). However, a new problem arises at this stage: indirect protein quantification methods, usually developed from the determination of the nitrogen content of the sample and its multiplication by a conversion factor, often overestimate the protein content of algae, due to the high presence in algae of non-protein nitrogenous substances such as pigments which can interfere and exaggerate the protein content (15). Moreover, the nitrogen-based conversion factor of 6.25 usually employed for protein quantification overestimates the real protein content by 43% according to Samarathunga et al. (20). Angell et al. (21) proposed the use of different nitrogen-protein ratios for each algal family, being 5.1 for red algae, 4.49 for green algae, and 4.56 for brown algae, respectively. Therefore, the sum of the amino acid residues after hydrolysis is often the chosen method (20).

### 2.2. Protein types in algae

Seaweed proteins are mainly glycoproteins, lectins, mycosporine-like amino acids, and phycobiliproteins (20). Phycobiliproteins are also contained in microalgae, such as *Spirulina platensis* (23). Glycoproteins are the main group of proteins in marine algae and are contained within the cell wall, where they perform physiological functions (20). These proteins are responsible for covalent binding to the complex carbohydrates of algae by glycosylation. Arabinogalactans and hydroxyproline-rich glycoproteins stand out within this group (20). Lectins, also called agglutinins, are proteins that have the affinity to bind to simple carbohydrates. Within this group, lectins binding to mannose, galactose, N-acetyl-glucosamine, fucose, and sialic acid stand out according to their affinity to bind to different sugars (20).

Another group of proteins in algae is constituted by a group of secondary metabolites called mycosporine-like amino acids. These metabolites have low molecular weight, are amphoteric molecules, and their main function is to absorb ultraviolet radiation in the wavelength range 310–365 nm (20). Finally, phycobiliproteins, proteins located in the stroma of the chloroplast, are the main proteins of red algae, responsible for a higher protein content of these algae than green and brown algae (15). Within this group, phycoerythrin, phycocyanin, allophycocyanin, and phycoerythrocyanin can be highlighted as the main compounds form seaweeds (20), while phycoerythrocyanin, phycocyanin, phycoerythrin, and allophycocyanin are the main phycobiliproteins obtained from microalgae (23). They have non-toxicity benefits, bright color, and high-water solubility, hence very acceptable for food applications (23). Besides, they are further associated with excellent health benefits, involving reducing nerve damage anti-inflammatory, anti-allergic, antioxidant, and anti-tumor properties (24).

One of the main advantages related to both seaweed and microalgae protein content is the high proportion of essential amino acids (EAA). Their content in EAA is much higher than those proteins from terrestrial plants (17). The most abundant amino acids in seaweed are glycine, alanine, arginine, proline, glutamic acid, and aspartic acid, while they contain low levels of tryptophan, cysteine and lysine. Previous works have shown that the EAA content in some algal species, such as *Caulerpa lentillifera* and *Ulva reticulata* accounted for almost 40% of total amino acids and the essential amino acid profiles approached to those from egg and soy protein (20). Therefore, combining seaweed intake with other protein-enriched foods could be considered an optimal approach for high-quality protein intake (14). Nevertheless, seaweed proteins usually present low digestibility, caused by the high content of anti-nutritional molecules such as some polysaccharides or tannin derivatives (14). Another advantage, specifically in the microalgae proteins is the stability and emulsifying capability, similar to those of traditional emulsifiers for proteins obtained for some microalgae species such as *Chlorella vulgaris* (23), as well as the content of microalgal-derived bioactive peptides are also known for their antihypertensive, immune regulatory, antibacterial, and antiallergic properties (23).

### 2.3. Algae protein extraction

Primarily, the lower efficiency of protein extraction from algae is due to the more complex and heterogeneous structure of the cell walls of marine algae than those of terrestrial plants (20). Algal cell walls are composed of mixtures of sulfated and branched polysaccharides that are associated with proteins. Therefore, treatments are needed to degrade these structures to favor the extraction of proteins (15). No method for determining the protein content in protein extracts obtained from algae is accurate, since extrinsic factors such as protein extraction techniques, protein quality and the presence of other compounds can significantly interfere with the results (15). It also affects algal species and preservation techniques (20).

Regardless of the technique used, protein recovery from seaweed follows two different steps: Cell disruption and protein extraction, and a subsequent protein concentration process (15). Cell disruption aims to rupture cell walls to release the intracellular fraction of biological samples, including free proteins and those attached to the cell wall (14). Among the most used cell disruption methods are mechanical crushing, osmotic shock, alkaline treatment, freeze-thawing or ultrasonic sonication (20). The alkali extraction and acid precipitation method is the most commonly used protein extraction method (25). The protein extraction process is performed once the cell walls have been ruptured. The most used extraction methods currently employed for this purpose include solid-liquid extraction, enzyme-assisted extraction, electric pulse, high hydrostatic pressure extraction, ultrasound-assisted extraction, and microwave-assisted extraction (14). All these techniques improve the mass transfer rate, increasing the interaction between solvent and solute, which helps to reduce the extraction problems caused by the matrix complexity (18).

After extraction, the proteins obtained from seaweed must be purified to remove other co-extracted components, such as polysaccharides, minerals, and phenolic compounds, while increasing the protein content in the final product (13). Protein purification methods are based on molecular charge and size differences, and the most used methods are ultrafiltration, ion exchange chromatography and dialysis (14). Subsequently, the obtained extracts must be dried, followed by a protein concentration stage, achieved by acid precipitation, solvent, electrolyzed and isoelectric water, filtration, hydrolysis, and chromatography (15).

More interesting is the option of extracting protein from the microalgae residue that remains after previously extracting the fat, since the content of this residue represents about 70% of the residual biomass (25). As mentioned above, no suitable method can be applied to all marine algae, so at each stage, the method must be adapted to the type of algae used and the type of protein product to be obtained (20). Depending on the degree of refining, protein products obtained from microalgae can be classified as whole-cell proteins, protein concentrates, isolates, hydrolysates, and bioactive peptides (26).

For this case, dried algal biomass must first be dispersed in an extraction solvent before cell disruption. Cell disruption methods used in microalgae can be classified as physical (drying, sonication, and pulsed electric field), mechanical (bead milling, homogenization and chemical/biological (acid, base, and enzymes) (26). Once the microalgae cells have been disrupted, cell debris must be removed and the obtained extracts must be concentrated to produce protein concentrates or isolates via precipitation and/or diafiltration (26).

## 3. Algae as an ingredient in protein meat alternatives

Algae are a source of techno-functional and bioactive compounds including hydrocolloids, pigments, vitamins, minerals, fatty acids, antioxidants, proteins, and bioactive peptides, among others (27, 28). During the protein transition of animal to vegetable proteins, both microalgae and macroalgae have been widely recognized as a nutritious and sustainable options due to their high concentration of proteins (over 50% of dry weight) and nutritional value (29, 30). However, in order to include proteins in food systems they need to present adequate techno-functional properties. Previous revisions have concluded that microalgae proteins possess emulsifying, foaming, gelation, and solubility properties similar or even higher compared to other vegetable proteins such as soy, frequently added as an ingredient in meat formulations (29, 31). Notably, the emulsifying capacity and stability of proteins extracted from *Chlorella vulgaris* are comparable to the emulsifying capacity and stability of commercial emulsifiers (25). Nevertheless, the use of algal proteins to produce meat analogs using purely this protein as a raw material represents a significant challenge for the food industry (32). In fact, the general approach has been using the algae protein, or the whole algae, as an ingredient replacer in meat alternatives by replacing soy protein. The main difficulty to produce meat alternatives based on microalgae lies in the early stage of research and development of successful techniques to texturize algal biomass, the need to remove the undesirable odors and colors usually provided by algae, and the lack of improvements in the cultivation processes to accomplish an efficient scaling-up (29).

The key step to producing meat analogs is the selection of the technology to develop the textural profile that resembles meat fibers (30). Extrusion stands as one of the most used technologies to produce meat analogs. During extrusion, protein ingredients are texturized into fibers, shreds, chunks, bits, granules, or slices to contribute to the textural properties of the final product (33). Meat analogs can be produced by low and high moisture extrusion (HME), the latter being preferred due to the formation of fibrous structures with a greater resemblance to real meat muscle (34). The most common raw materials to produce extruded meat analogs are soy, pea, and gluten but other protein sources can also be used (35). The substitution of these raw materials, specifically soy, with algal proteins, has been barely evaluated in a few studies. In this regard, Grahl et al. (36) studied the effect of substituting soy with 10%, 30%, or 50% spirulina (*Arthrospira platensis*) as an entire biomass to produce textured vegetable proteins through extrusion. The effect of extrusion temperature (140°C to 180°C), screw speed (from 600 rpm to 1,200 rpm), and feed moisture (57% to 77%) was also evaluated. The extrudates were analyzed by a trained sensory panel and instrumental texture and shear force methods. The incorporation of spirulina yielded extrudates with black color and an intense flavor with earthy notes and an algal odor. High concentrations of spirulina (50%) negatively affected extrudates' fibrousness, firmness, and elasticity. Both, the content of spirulina and feed moisture, during extrusion played the most significant role in the sensory and textural properties of the final product, while the temperature and screw speed affected to a lesser extent. However, high temperature and screw speed levels were needed for texture formation. So, keeping the moisture low, and increasing the screw speed and temperature, allowed the authors to obtain more fibrous, elastic, firmer, and layered products using substitution levels of 30 and even 50%. In fact, higher moisture levels resulted in a juicier and softer mouthfeel with a moist appearance. This study proved that it was possible to partly substitute soy proteins, typically used to produce textured proteins, with whole spirulina biomass. However, the darker color of the highly substituted meat analogs should be one of the aspects to overcome for greater acceptability.

Caporgno et al. (37) produced a fibrillary textured meat analog using HME with the heterotrophically cultivated *Auxenochlorella protothecoides* microalgae as a partial substitute for a soy protein concentrate. The microalgae reduced the texturization at high concentrations (50%) probably due to limited access to the intracellular proteins during the process and to the lubrication effects triggered by the high fat content in microalgae biomass that decreased the shear forces during extrusion and negatively affected the protein texturization. Consistently with Grahl et al. (36) findings, Caporgno et al. (37) observed that the reduction in the feed moisture level allowed to balance the negative effects caused by higher concentrations of microalgae biomass. According to the authors, it was possible to obtain extrudates without a significant detriment in their quality at a substitution level of 30% and a moisture level of 60%. Since the heterotrophic controlled conditions yielded yellow pigments in *Auxenochlorella protothecoides*, substituting soy proteins with the microalgae did not cause adverse colors. Moreover, the incorporation of microalgae enriched the nutritional profile of the extrudates by incorporating vitamins B (B1, B2, B3, and B6) and vitamin E which were retained after extrusion. The authors also evaluated dry extrusion but it was not possible to establish a constant process due to pressure and torque fluctuations. Further, the fibrous texture was significantly lost while increasing the concentration of microalgae biomass. Therefore, high-moisture extrusion was more suitable to produce soy-based meat analogs substituted with microalgae biomass.

Palanisamy et al. (38) evaluated the substitution of lupin proteins with Spirulina platensis to produce meat analogs using HME. The effect of Spirulina concentration (15, 30, and 50%), barrel temperature (145°C to 170°C), feed moisture (50 to 60%), and screw speed (500 to 1,200 rpm) were evaluated. Higher concentrations of Spirulina (30 and 50%) turned the color from yellow to dark green, decreased the cutting force indicating a decrease in the formation of fibrous structures, and led to lower cooking yields. Meanwhile, a lower concentration (15%) did not show significant differences in cutting force and cooking yields compared to the 100% lupin control. Similar to the results obtained by Grahl et al. (36) in soy-based meat analogs, increasing the temperature (175°C) and screw speed (1,200 rpm) led to higher cutting forces in Spirulina-lupin meat analogs which could be probably caused by higher protein denaturation. Therefore, it was concluded that desirable physical characteristics may be achieved by optimizing the extrusion parameters resulting in substitutions of up to 50% with Spirulina flour. Moreover, increased temperature and screw speed combined with low moisture may also lead to higher concentrations of bioactive molecules such as phenolic compounds and flavonoids, and consequently to higher antioxidant activity.

One of the most significant advantages of including algae and algal proteins in meat analog formulations would be the nutritional improvements related to these ingredients. Algae may help to increase the protein content of meat alternatives, however, it would depend on the level of substitution/supplementation and the protein concentration in the ingredients. For instance, commercial spirulina biomass and soy protein concentrates typically contain around 70% and 65%, respectively (36). Therefore, if spirulina is used to substitute 30 to 50% of the soy protein concentrate in meat-analog formulations, the protein content of the blend would increase up to 2.5 units compared to the 100% soy formulation. Similarly, when algae have been used to supplement meat formulations, an increment in the protein content of up to 1.2 units was found when 2.5% to 5.6% of Nori seaweed (39% protein in dry basis) were added, but when seaweed with <12% protein was supplemented, no significant differences were observed (39, 40). Increasing the protein concentration in the algae ingredients used to produce meat analogs will lead to higher increments of this nutrient in the final products. Moreover, studies have shown that algae allow not only the increase of protein content due to their high concentration, but also to improve the amino acid profile since they contain a good ratio of essential and non-essential amino acids and have high amino acid scores (41–43). Microalgae such as Nannochloropsis sp., Scenedesmus sp., and a chlorophytic polyculture consisting of *Schroederiella apiculata, Scenedesmus pectinatus, Tetraedrom minimum, Mesotaenium* sp., and *Desmodesmus* sp. had amino acid scores higher than 1 (44), which means that their proteins do not present any limiting amino acid, unlike proteins from other vegetable sources. However, the essential amino acid contents vary depending on the micro- and macro-algae species. De Bhowmick and Hayes (45) found significant differences in the limiting essential amino acids depending on the seaweed species. The limiting amino acids in the brown seaweeds *A. esculenta, F. serratus* and *F. vesiculosus* were L-tyrosine + L-phenylalanine, L-histidine, and L-leucine, respectively. The red seaweeds *P. palmata* and *A. taxiformis* had as limiting amino acids L-tyrosine + L-phenylalanine and L-cysteine + L-methionine, respectively. Finally, the limiting amino acids in the green seaweed *U. latuca* were L-cysteine + L-methionine. The amino acid scores, representing the relation between the limiting amino acid content in the test protein and the amino acid requirement pattern, were also different among species with values ranging from 0.101 to 0.883 (De 43). Balancing the essential amino acid content with a multiple algae mixture could improve protein quality (29). For instance, in a meat-emulsion model containing edible seaweeds, Sea Spaghetti helped improve the sulfur amino acid score while Nori improved the valine score (40). Combining these two seaweeds could lead to an increase in the amino acid score of both essential amino acids. In fact, complementing essential amino acids with a combination of sources is a common practice to improve the protein quality of vegetable proteins. Digestibility is the other parameter that together with the content of essential amino acids, determines the protein quality. It is a measurement that gives information about the bioavailability of proteins. Similar to the amino acid profile, studies have indicated that the alga species affects the digestibility of their proteins (46). In a revision done by Fu et al. (29), *Chlorella* sp. proteins had a higher digestibility coefficient (DC) compared to *Arthrospira* sp. (93.6% vs 88.2% compared to the DC of casein). Van de Walle et al. (46) reported *in vitro* protein digestibility (IVDP) values of Arthrospira platensis and *Chlorella* sp. ranging from 74 to 81% and 55–79%, respectively, but differences were found both depending on the species and the protocol used to quantify IVDP. The protein bioavailability may also be determined by the measurement of the protein efficiency ratio (PER), biological value (BV), and net protein utilization (NPU) which were also affected by the microalgae species (29). The lower digestibility of microalgae has been associated with the robust cell walls that limit the accessibility of digestive enzymes to proteins, and to the matrix composition when they are included in food products (46). On the other hand, De Bhowmick and Hayes (45) found similar IVDP values ranging from 0.77 to 0.82 when six seaweed comprising brown, red and green species were analyzed. After relating the digestibility with the limiting amino acid score, the protein digestibility corrected amino acid score (PDCAAS) is obtained, which is one of the most important parameters used to characterize the protein quality. This value ranged from 0.15 to 0.69 in the seaweed samples evaluated by De Bhowmick and Hayes (45). Authors found that these values were lower than the PDCAAS of soy (0.91) and rice (0.81), but the PDCAAS of *Palmaria palmata* (0.69) and *Fucus serratus* (0.63) were similar to chickpeas (0.62–0.65). Moreover, during the production of Spirulina-lupin meat analogs using HME, Palanisamy et al. (38) observed that increasing concentrations of Spirulina significantly reduced the IVPD from 82% to 78%, 76%, and 74% when control was compared to the samples substituted with 15%, 30%, and 50% Spirulina, respectively. Since the digestibility is affected by the reduced accessibility of enzymes to proteins, disruption of microalgae cell walls by physical, chemical, and enzymatic methods could improve this parameter as reviewed by Van de Walle et al. (46). Moreover, these pretreatments used to increase the accessibility of proteins, could also improve their ability to contribute to forming cross-links and fibrous structures during texturizing processes such as extrusion. Including algae in meat formulations has also been related to increasing the concentrations of n-3 polyunsaturated fatty acids (PUFA) associated with significant health benefits (41). Moreover, algae are a good source of minerals and nutraceutical compounds such as phenolic compounds and flavonoids with high antioxidant activity, so their incorporation may lead to producing functional meat alternatives with excellent nutritional value (27, 29, 38).

Although the use of algae proteins in meat alternative formulations requires more research, the acquired knowledge of using these ingredients in meat formulations could help in the development of processed meat analogs. For instance, purified seaweed compounds such as carrageenan or alginate have been used in meat product formulations for many years due to their outstanding techno-functionality. Carrageenan serves as a gelling, thickening, and stabilizing agent in several processed meats including sausages, burgers, meatballs, and corned beef, while alginates are used as binders and fillers in restructured meat (47, 48). In fact, iota carrageenan has also been added as an ingredient in producing lupin-based HME meat analogs containing Spirulina due to its ability to improve texturization (38). Although isolated compounds have specific technological functionalities, using the whole algae presents several advantages such as reducing the time, costs, and environmental impact of refining processes. They can also incorporate a higher diversity of techno-functional compounds and nutrients into the formulation (47). The use of semi-refined ingredients obtained from algae has also been evaluated. For instance, semi-refined carrageenan maintains its ability to enhance meat products' textural and structural properties but is less cost-effective than the refined counterpart (49).

Table 1 summarizes several applications of seaweed and microalgae in meat formulations and examples of the effect of these ingredients on the techno-functional and organoleptic properties of final products. It can be observed that the incorporation of algae, especially as whole biomass, may result in accomplishing several technological functionalities due to the variety of compounds that it contains. In general, algae and its extracts are included in concentrations up to 6% to attain these functionalities although the study performed by Cox and Abu-Ghannam (50) evaluated the substitution of meat with *Himanthalia elongata* from 10 to 40% w/w. The use of seaweed as a salt substitute is one of the applications that has recently gained attention due to the worldwide need to reformulate food products to contain less sodium. This effect can be achieved due to the high content of minerals of algae which may reduce the intake of Na while increasing the intake of other minerals (51) and its umami flavor attributed to the presence of amino acids such as glutamic acid and aspartic acid (52). In fact, glutamic acid is the predominant amino acid in several micro- and macro-algal species (40–44, 53). Replacing salt is a challenge for the meat industry since besides contributing to flavor, the salt is associated with water binding capacity of meat proteins, modulation of enzymatic activity, firmness, and enhancement of microbiological safety (54). In this sense, the benefit of reducing sodium with seaweed extracts is based on its capacity to overcome the techno-functional problems associated with low-salt meat formulations. For instance, the edible seaweeds sea tangle (*Laminaria japonica*), sea mustard (*Undaria pinnatifida*), hijiki (*Hizikia fusiforme*), and glasswort (*Salicornia herbacea* L.) promote the fat and water binding properties which improve the emulsion stability and reduces the cooking losses in meat products (55, 56). Some studies which have evaluated the reduction of salt with seaweed, have also evaluated the reduction of fat in meat formulations due to the properties of these ingredients as structuring agents. The emulsifying and strengthening of water and oil retention capacities by the addition of seaweed Sea Spaghetti (*Himanthalia elongata*) and *Undaria* sp. led to increasing firmness, hardness, and elastic properties of reformulated meat products, attributed to the presence of alginates and dietary fiber (57, 58). Ingredients based on seaweed extracts are already in the market to replace salt in food formulations (51). An example of this commercial ingredient is Algysalt^®^, a seaweed mixture containing *Lithothamnium calcareum, Laminaria, Enteromorpha, Ascophyllum nodosum, Palmaria palmata, Fucus vesiculosus, Himanthalia elongata, Laminaria saccharina, Ulva lactuca, Chondrus crispus, Porphyra umbilicalis, Palmaria/Porphyra/Ulva*, and *Undaria pinnatifida*. Substitution of NaCl with Algysalt^®^ and sodium-free salts (KCl, MgCl2 and CaCl2) was studied by Triki et al. (59) in sausages. They found increased hardness and lower cooking losses compared to sausages prepared with a mixture of sodium-free salts. During the sensory evaluation, the general acceptability did not show significant differences from those produced with the sodium-free salt mixture. Moreover, the microbiological quality was not affected by replacing salt with both the seaweed extract or sodium-free salt mixture, demonstrating that both substitutes have similar preservative effects as NaCl (59). Cox and Abu-Ghannan (50) found significant reductions in the bacterial counts of beef patties supplemented with *Himanthalia elongata* after 30 days of storage compared to the control. In fact, there was no bacterial growth when the concentration of seaweed was equal or higher than 20%. However, the microbiological stability could be dependent on the seaweed used. Vilar et al. (60) produced frankfurters substituted with 1% edible seaweeds (*Porphyra umbilicalis, Palmaria palmata, Himanthalia elongata*, and *Undaria pinnatifida*) and found that samples containing *U. pinnatifida* presented significantly higher total viable counts during storage compared to the control and the other seaweed samples indicating a lower shelf life when this seaweed is used. The antimicrobial potential of seaweed and seaweed extracts has been reviewed and related to the great variety of bioactive metabolites such as polysaccharides, polyunsaturated fatty acids, phlorotannins, phenolic compounds, lectins, alkaloids, terpenoids and halogenated compounds (48, 61). The reduced microorganism counts even at low salt concentrations could be related to the activity of these compounds. A comparison of products of the Australian market found that only 4% of the plant-based meat analogs were low in sodium (62). Therefore, reducing sodium with seaweed could be an attractive alternative since it could also enhance the texture and shelf-life of meat analogs. Moreover, since some consumers perceive a lack of naturalness in meat analog formulations (63), the evaluation of seaweed to substitute texturizing agents and chemical preservatives in these products, could result in a natural alternative to obtain cleaner labels.

**Table 1:** Selected studies reporting the applications of whole seaweed/microalgae and extracts in meat products.

**Application**	**Meat product**	**Seaweed or microalgae**	**Usage level**	**Effect of algae in technological and organoleptic properties**	**References**
Salt reduction	Gel-emulsion systems	*Himanthalia elongata, Undaria pinnatifida*, and *Porphyra umbilicalis*	2.5% and 5%	↑ water and fat binding (except Nori at 2.5%) ↑ hardness and chewiness; ↓ springiness and cohesiveness Color affected by seaweed type	Cofrades et al. (39)
	Frankfurters	*Porphyra umbilicalis, Palmaria palmata, Himanthalia elongata* and *Undaria pinnatifida*	1%	↓ 50% salt and 21% fat Darker color; ↓ liking of appearance ↓ hardness and chewiness Different volatile and sensory profiles	Vilar et al. (60)
Structuring agent	Pork meat batter formulations	*Himanthalia elongata*	3.4%	Replacement of animal fat with olive oil by ↑ the water/oil retention ↑ hardness and elastic modulus	Fernández-Martín et al. (57)
	Low-fat pork burgers	*Undaria* sp.	3%	↑ burger yield ↑ hardness and chewiness ↓ shrinkages compared to burgers produced with other emulsifiers (milk protein concentrate)	Nagai et al. (58)
	Low fat, low-salt beef patties	*Undaria pinnatifida*	3%	↑ binding properties ↓ thawing loss, and cooking loss of water, fat and ash ↓ saturated fat by replacement of pork backfat with olive oil	López-López et al. (64)
	Canned barbel fish burgers	*Chlorella minutissima, Isochrysis galbana, and Picochlorum sp*.	0.5% or 1%	Better texture and sensory properties ↑ swelling, water/oil holding capacities, and antioxidant activity ↓L^*^, a^*^ and b^*^ values ≈ color acceptability vs control	Ben Atitallah et al. (65)
	Canned carp and barbel fish burgers	*Spirulina platensis*	1%	Better texture and sensory properties ↑ swelling, water/oil holding capacities due to dietary fiber ↑ antioxidant activity ↓ yellowness and ↑ greenish color; ≈ color acceptability vs control but ↓ with higher levels (1.5%) No mold or foodborne pathogens detected up to 8 months at 4°C	Barkallah et al. (66)
	Fish surimi restructured fingers	*Ulva intestinalis* powder (UIP), and isolated sulphated polysaccharide (USP)	UIP at 2.77 g per kg and USP at 0.5 g per kg	Stable textural properties for 6 months (control became harder) Acceptable sensory attributes USP fingers: ↓ cooking loss, juicier texture and ↑ acceptability	Jannat-Alipour et al. (67)
Antioxidant activity	Meat emulsion formulations	*Himanthalia elongata, Undaria pinnatifida*, and *Porphyra umbilicalis*	5.6%	↑ antioxidant capacity due to soluble polyphenolics ↑ antioxidant activity in *H. elongata* samples	López-López, Bastida et al., (40)
	Reduced nitrite turkey meat sausages	Fucoxanthin purified from *Cystoseira barbata*	0.01%, 0.02% and 0.04%	↑ antioxidant properties ↓ nitrites from 150 to 80 ppm ↑ color stability during 15 days in refrigerated storage ↓ lipid peroxidation, ↑ oxidative stability	Sellimi et al. (68)
	Pork liver pâté	Extracts from *Ascophyllum nodosum, Fucus vesiculosus* (FV), and *Bifurcaria bifurcata (BB)*	500 mg per kg	↓L^*^ values in FV and BB batches; ↑a^*^ and b^*^ after 180 days storage; no sensory evaluation ↑ protein content Similar protection than tert-butyl-4-hydroxy-toluene (BHT)	Agregán et al. (69)
	Raw ground pork meat	*Haematoccocus pluvalis* extract rich in astaxanthin	0.30 or 0.45 g/kg	↓ lipid oxidation ↑ color stability (↑a^*^), ↑ color acceptance ↑ acceptability by consumers after storage No effect in microbiological or protein oxidative stability	Pogorzelska et al. (70)
	Chinese-style sausages	Polysaccharides in *Spirulina platensis*	0.5%	↓ lipid peroxidation during refrigerated storage at 4°C for 24 days Redness (a^*^) maintained during storage, ↑ overall acceptability vs control ↑ aroma, flavor and overall acceptance during storage No effect in microbiological stability	Luo et al. (71)
	Liposome meat system	Extracts of Phycobiliproteins from *Porphyra haitanensis*	1%, 3% and 5%	↑ radical scavenging effect and Fe^2+^ chelating activities ↓ lipid peroxidation and organoleptic alteration ↑ essential amino acid content	Chen et al. (72)
	Rainbow trouts	*Chlorella vulgaris* and *Spirulina platensis* alcoholic extracts	0.1%	Controlled peroxide value, thiobarbituric acid and free fatty acid ↑ green color (specially *Spirulina platensis*); no negative effect on color acceptability Maintained color, odor, texture and taste during 16 days of refrigerated storage	Taghavi Takyar et al. (73)
Source of nutrients	Cutlets, meatballs, quenelles, and griddle sausages	*Fucus, Cystoseira*, and *Laminaria*	2%	*Cystoseira* or *Fucus* were good Se and I sources *Laminaria* was not recommended due to very high I and Se contents	Kryzhova et al. (74)
	Beef patties	*Himanthalia elongata*	10 to 40%	↑ dietary fiber, total phenolic content and antioxidant activity ↓ cooking loss ↑ tenderness by 50% vs. control ↓ sensory acceptance ↓ lipid oxidation and microbiological counts	Cox and Abu-Ghannam (50)
Replacement of soy proteins	Turkey breast formulation	*Spirulina* and *Chlorella* proteins	1%	↓ soy allergens ↑L^*^ and ↓a^*^ (greenish color); no sensory evaluation ↓ hardness vs. soy	Marti-Quijal et al. (42)
	Chicken rotti	*Spirulina* and *Chlorella* proteins	1%	↓L^*^, b^*^, and a^*^ (higher greenness); ↓ sensory acceptability ↓ hardness, cohesiveness, chewiness and gumminess vs. soy ↓ acceptability and preference in sensory analysis	Parniakov et al. (43)
	Fresh pork sausages	*Chlorella* *and* *Spirulina* proteins	1%	↓L^*^ and a^*^ vs. soy; *Spirulina* ↓b^*^; no sensory evaluation ↓ hardness, elasticity, gumminess and chewiness; no changes in cohesiveness *Spirulina:* ↑ total, essential and non-essential amino acid contents	Marti-Quijal et al. (75)
Replacement of phosphates	Frankfurters	Dietary fiber extracted from red seaweed	1%	No change in emulsion stability No differences in hardness, resilience, and springiness but ↓ chewiness ↑L^*^ and b^*^; ↓a^*^; ↓ interior color scores ↓ color and flavor acceptability but good scores in juiciness and homogeneity ↓ lipid oxidation during storage	Yuan et al. (76)

Although including seaweeds in meat yields significant nutritional and texture-related benefits, their incorporation can present sensory disadvantages such as the change to darker or greener colorations, the incorporation of non-typical flavors, and dry mouthfeel. These changes affect the acceptability of meat products containing algae that is probed by the lower scores obtained in the parameters of color, flavor, and overall acceptability during sensory analysis (55, 77, 78). These challenges should also be addressed if algae is included in meat alternative formulations.

The reduction of undesirable odors has been evaluated by deodorization techniques but also by avoiding the synthesis of the responsible volatile compounds. The deodorization of *Arthrospira platensis* biomass was evaluated by Cuellar-Bermúdez et al. (79) using ethanol, acetone, and hexane. The most effective solvent to remove odoriferous compounds was ethanol. After ethanol extraction, the spent cake presented the best sensory results among samples, due to the lower fishy odor detected by panelists. Since different algae have different aromatic profiles related to different aromatic compounds (80), the solvents used to deodorize the matrix would be different depending on the polarity of the aromatic compounds intended to be removed. The concentration of undesirable aromatic compounds has also been reduced by the addition of nitrogen in the growing media or by controlling the growing time during microalgae cultivation (29). Due to the significant contribution of lipid-derived aromatic compounds, it is important to control lipid oxidation in algae products to ensure good flavor quality (80). The reduction of lipid oxidation may be attained by the algae antioxidants (Table 1). *Himanthalia elongata* probed to reduce around 45% the oxidation of beef lipids during 30 days of storage (50). Also, extracts from *Ascophyllum nodosum, Fucus vesiculosus* and *Bifurcaria bifurcata* seaweeds used in pork liver pâté showed a similar degree of protection against oxidation compared to the synthetic antioxidant tert-butyl-4-hydroxy-toluene (BHT) (69). Moreover, the desired flavor profile would also depend on the type of meat product. For example, in fish-based products such as surimi, the incorporation of the green seaweed *Ulva intestinalis* did not negatively affect the flavor or odor scores during sensory analysis (67). Also, in rainbow trout filets, the addition of *Chlorella vulgaris and Spirulina platensis* presented better odor and taste liking scores during storage, in comparison to control samples (73). The potential of microalgae to serve as a natural flavoring and coloring agent in fish-based products has been noted, however it is important to consider that high concentrations may lead to undesirable taste caused by lipid oxidation, and minerals with pro-oxidant activity or metallic off-flavors (65, 66). Based on the positive effects of algae in the taste and odor of fish products, it could be assumed that algae that carry fishy notes could be suitable to provide this flavor to fish analogs. However, studies to confirm the more suitable species and levels of incorporation- for this purpose are needed. Although the consumer opinion on algae meat-analogs is positive since they recognize this protein source has nutritional and environmental advantages, they have low taste expectations (81), which could negatively affect the buying decision. More studies to reduce off-flavors in raw materials and to improve the flavor of algae meat-analog formulations are needed to upgrade the opinion of consumers toward these new products.

In order to reduce the green and dark colorations associated with these in meat-algae alternatives, the change of cultivation conditions could be a feasible option since some microalgae produce light-yellow biomass when they are grown under controlled heterotrophic conditions, because of the accumulation of pigments as carotenoids (37). This color would be more compatible with some meat formulations such as pork, chicken, or turkey, and could help to eliminate the consumer dislike of the visual appearance. Another alternative could be the utilization of algae with lower chlorophyll contents. For example, Schüler et al. (82) developed a chlorophyll-deficient mutant of *Chlorella vulgaris* with decreased chlorophyll contents of 80% and 99% compared to the original strain, offering yellow and white colors, respectively. However, the best-growing conditions for both mutants were observed under a heterotrophic environment, while under light conditions only the yellow mutant grew, increasing its pigment concentration. But nevertheless, the inclusion of mutants could be rejected by the market, so finding wild algae with lower chlorophyll contents or changing the growing conditions seems like the better alternative to overcome the color-related problems.

The increments in fiber due to the incorporation of seaweed have been related to a dry mouthfeel sensation in the final product, especially when its incorporation is accompanied by fat reductions. For example, Jiménez-Colmenero et al. (77) proposed the inclusion of konjac glucomannan as an animal fat replacer in a reduced/low-fat and low-salt frankfurters formulation and found that the texture acceptability was not significantly affected when the fat was replaced with konjac glucomannan and Sea Spaghetti (*Himanthalia elongata*) but when the overall acceptability was evaluated, frankfurters containing seaweed recorded lower values attributed to the products becoming slightly dry and presenting seaweed-like off-flavors. On the other hand, Choi et al. (55) did not observe significant differences in the tenderness, juiciness, and overall acceptability during sensory evaluation of reduced-salt frankfurters added with 1% of sea tangle or sea mustard, compared to the control. In this study, the frankfurters were not reduced in fat which could contribute to the juicy mouthfeel. However, tenderness and juiciness were reduced when hijiki and glasswort seaweeds were used. These results could be related to the lower emulsion stability and higher cooking losses that these seaweeds presented during the study. Therefore, the selection of algae with adequate techno-functional properties must be confirmed to obtain the desired results. Moreover, if a reduced-fat formulation incorporated with fiber-rich algae is desired, the exploration of other fat replacers providing a creamy and wet mouthfeel related to fats but without increasing the caloric content could be used to compensate for the unwilling dry sensation. This is especially relevant in meat-alternative formulations since many ingredients need to be hydrated so the risk of dryness sensation is higher. Ingredients with a combination of high-water holding capacity, emulsifying properties, and wet mouthfeel sensation are desired to overcome the dryness problem.

## 4. Algae protein as a source of biofunctional peptides

Even though the first attempts at the utilization of algae were mainly focused on the polysaccharide obtention like carrageenan, the remarkable protein contents present in seaweeds and microalgae have opened a new alternative to algae uses. Apart from protein extraction and purification for supplementation or replacement purposes, the generation of bioactive peptides is recently in the spotlight due to the functional properties attributed to these molecules. Bioactive peptides are short-chain amino acids usually comprising 2–20 amino acids, even until 40 amino acids which remain encrypted within the parent protein structure and inactive until they are released showing then, the positive health outcomes (83, 84). The sequence and the composition of the amino acids present in the chain determine their biological activity (85). They can act as neurotransmitters, hormones, or antibiotics to produce positive health effects by binding to specific receptors or interacting with target cells (86). Although obtention of bioactive peptides can be achieved by fermentation processes (endogenous and exogenous microbial enzymatic action), food processing and gastrointestinal digestion of protein using several proteolytic enzymes use to being the preferred techniques to produce them (87). Enzymatic hydrolysis is low-cost, environmentally friendly, non-toxic, and provides good yields and *in vitro* bioactivities since peptides suffer less structural damage (88). Several protein-rich sources and byproducts have been subjected to the production of bioactive peptides such as milk, meat, fish, egg, legumes, or cereals (83). In the case of algae, protein biomolecules, as previously mentioned, are usually associated with polysaccharides in the cell wall, which limits protein extraction and makes necessary a pretreatment to release the proteins and peptide obtention. The pretreatments can include mechanical, physicochemical, and biological techniques. The mechanical processes such as shear stress, ultrasonication, or microwave radiation help to disrupt the cells allowing if it is the case the application of acids, alkalis, or organic solvents treatments or the use of microbes and enzymes to recover the protein and peptides (85). The application of enzyme-assisted extraction technology, combined sometimes with other techniques, increases the process yield and favors the generation of hydrolysates with less cell structural damage and it is the most popular technique in the extraction of bioactive peptides (85). Nevertheless, the application of novel methods such as pulse electric field processing or subcritical water extraction is gaining popularity in the extraction of algal proteins (28, 89–91).

In general, in the enzyme-assisted extraction cellulases, xylanases and proteases like alcalases or pepsin are commonly utilized enzymes to disrupt the cell wall to liberate the protein from polysaccharides (92), while different proteases such as pepsin, trypsin, elastase, papain, bromelain, chymotrypsin, alcalase have been employed in the protein hydrolysis both microalgae such as *Chlorella* sp or Spirulina (90) and macroalgae from genera *Ulva, Porphyra, Palmaria, Undaria* or *Laminaria* (92, 93). Once the peptides have been produced, the purification, concentration, and identification are the following steps. Membrane separation, size exclusion chromatography, or capillary electrophoresis are the general methods to purify the peptides (85, 94). Finally, the bioactive peptides require to be identified, generally by means of liquid chromatography-tandem mass spectrometry (LC-MS/MS) or ultrahigh performance liquid chromatography-tandem mass spectrometry (UPLC-MS/MS) (94).

In recent years, several reviews have been published related to the extraction and obtention of peptides from algae with biological activities including antioxidant, antihypertensive, anti-inflammatory, immunomodulatory, anticancerigenic and antimicrobial effects (85, 89, 90, 95, 96). Many of the published efforts have been focused on hypertension treatment, generally by the inhibition of angiotensin-converting enzyme ACE-I and renin activities by algae (95, 97, 98). Hypertension is a risk factor in cardiac-related diseases, which treatment is usually based on following a healthy diet and some drugs. The renin-angiotensin-aldosterone system is involved in the regulation of blood pressure by releasing renin and angiotensin (89). When blood pressure decreases, the renin enzyme is liberated, acting this enzyme on angiotensinogen, resulting in the conversion of angiotensin I. This compound can be transformed into angiotensin II, a potent vasoconstrictor, by the action of angiotensin-I-converting enzyme which leads to vasoconstriction (99). In general, peptides with aromatic or aliphatic amino acids (such as proline, tyrosine and phenylalanine) at the C-terminal or peptide chains with valine/isoleucine amino acids at the N terminal have been reported to have the anti-hypertensive effects (100, 101). In the market, there are some commercial algae peptides with functional claims (102, 103). Wakame jelly, Nori S Peptide, or Mainichi Kaisai Nori are some commercial peptides obtained from Undaria pinnatifida (wakame) or Porphyra yezoensis (nori), respectively, focused on the antihypertensive function (104, 105). Even hydrolysate from *Palmaria palmata* protein (4%) with *in vitro* renin inhibitory properties have been successfully tested in wheat bread without hardly affecting the texture sensorial properties but keeping the renin inhibitory bioactivity (106).

The antioxidant properties of some algal peptides could make them an excellent additive to consider in the formulation of meat products, where lipid and protein contents make them susceptible to oxidation, affecting the organoleptic characteristics and shortening the shelf life (101). However, despite the antioxidant properties found in different algal species attributed to protein hydrolysates, as well as, polyphenols or oligosaccharides (107), to our knowledge, no specific studies have been conducted in the development of health-promoting functional meat products or analogs by addition of peptides. Gonçalves et al. (108) studied the effect of polycaprolactone nanofibers added with biopeptides obtained from Spirulina hydrolyzed with Protemax 580 L enzyme as an active packaging in the preservation of chicken meat cuts during cold storage. The application of peptide nanocomposites reduced the degradation of the sample exhibiting lower TBARs and N-BVT values due to the antioxidant and antimicrobial activity of peptides.

The scarce research on the food application of biopeptides could be attributed to certain challenges that are still pending further study. First, the functional properties of algal peptides are usually reported on *in vitro* assays, but it is unknown whether *in vitro* activities are correlated with *in vivo* activities (109). Some food processing techniques such as thermal treatments or fermentation could affect the bioactive peptides, as well as the protein, and reduce the biofunctionality (92). Moreover, the biopeptides must remain through the gastrointestinal digestion pathway to reach the target site in optimal conditions to exert their activity (83). For this purpose, the encapsulation by spray-drying of peptides in different matrices such as maltodextrin, gum arabic, and gelatin, besides preventing their degradation, could also avoid the bitterness associated (83, 110). Biopeptides have a bitter taste, which can be translated into a functional food product. According to Sarker (83), this characteristic seems to be associated with the hydrophobic property of the amino acids, increasing the bitterness the more hydrophobic groups there are at the C-terminals (111).

The second challenge to face would be the routine industrial peptide production from algae protein since algae protein contents depend on factors such as location or harvest time which complicates the standardization of the protein extraction process (92). Purification of peptides and obtaining them in sufficient quantities is a complex and expensive process (84). The large-scale production of peptides also makes chromatographic techniques for purification unsustainable. The use of immobilized enzyme systems in an enzyme membrane bioreactor or ultrafiltration coupled with electro-dialysis should be the chosen techniques in industrial scenarios (28, 84).

Apart from that, several toxic compounds have been found in algae, which demands a purification of the peptides to assure the safety of food products, even though some concerns about allergenicity should be taken into account (101). But also, the purification of peptides from hydrolysate might reduce the potency due to the removal of synergistic peptides present in the original hydrolysate (90). In this sense, the growth of algae under controlled conditions could be an option but increasing the costs of the peptide obtention.

There is no doubt about the promising future of the use of peptides from algae sources in human supplementation and the development of functional foods, but further research is needed to address the feasibility and safety implications to offer the consumer functional and affordable meat alternatives without demeriting the sensorial properties. In this context, the application of *in silico* methods and computational modeling becomes an invaluable approach to predicting potential bioactivities, which could save time and money, facilitating the supply of stable and quality peptides as an additive (83, 112). Additionally, marine environment is a source of diverse compounds, affecting aquaculture industry then by the geographical, anthropogenic and seasonal factors. Besides the beneficial aspects of algal use, it should be considered the potential presence of the toxic chemical compound from seaweed (i.e., prostaglandin A2 and prostaglandin E2), high levels of iodine and heavy metals and arsenic contamination as consequence of absorption processes (113).

## 5. Conclusion

In the search for newly promising and sustainable protein sources for the development of meat alternatives, algae seem to be the perfect match offering high protein contents, low growing requirements, high photosynthetic efficiency, and accomplishing the sustainable requirements with a low carbon footprint. However, protein extraction, purification, and concentration still represent outcoming challenges to producing a standard, homogeneous, safe, and bioavailable protein ingredient, especially when so many environmental and species factors affect the protein content and the presence of toxic or allergenic compounds. Algal protein can be used in the development of meat analogs using high moisture extrusion as texturizing technology, especially partially substituting soy proteins. The few published papers confirm that the production of fibrous texture is possible when adjusting extrusion process parameters. Moreover, the knowledge that has been gained in the use of whole algae and extracts in meat products may also be applied to develop meat analogs with lower salt and fat contents or to replace refined texturizing agents, synthetic antioxidants, or phosphates with algal ingredients which may help to improve their perception of naturalness. However, there are other aspects to consider in the use of algae such as the impact on the sensorial properties or the natural resistance of the consumer toward this ingredient. This initial dislike could be overcome if nutraceutical claims like the inclusion of antioxidant activity and bioactive peptides were introduced in the formulation of meat alternatives. Although the research about the use of algal in the development of meat alternatives is at an initial stage, promising results could be expected in the near future due to their diverse composition of bioactive and techno-functional molecules.

## Author contributions

JE-R: Conceptualization, Writing—original draft, Writing—review and editing. AM-P: Writing—original draft, Writing—review and editing. JR: Conceptualization, Supervision, Writing—original draft, Writing—review and editing. JL: Writing—original draft, Writing—review and editing. ES: Conceptualization, Supervision, Writing—original draft, Writing—review and editing.
